# *In Silico* Genome Comparison and Distribution Analysis of Simple Sequences Repeats in Cassava

**DOI:** 10.1155/2014/471461

**Published:** 2014-10-13

**Authors:** Andrea Vásquez, Camilo López

**Affiliations:** Molecular Biology Laboratory, Biology Department, National University of Colombia, Carrera 30 No. 45-03, Bogotá, Colombia

## Abstract

We conducted a SSRs density analysis in different cassava genomic regions. The information obtained was useful to establish comparisons between cassava's SSRs genomic distribution and those of poplar, flax, and *Jatropha*. In general, cassava has a low SSR density (~50 SSRs/Mbp) and has a high proportion of pentanucleotides, (24,2 SSRs/Mbp). It was found that coding sequences have 15,5 SSRs/Mbp, introns have 82,3 SSRs/Mbp, 5′ UTRs have 196,1 SSRs/Mbp, and 3′ UTRs have 50,5 SSRs/Mbp. Through motif analysis of cassava's genome SSRs, the most abundant motif was AT/AT while in intron sequences and UTRs regions it was AG/CT. In addition, in coding sequences the motif AAG/CTT was also found to occur most frequently; in fact, it is the third most used codon in cassava. Sequences containing SSRs were classified according to their functional annotation of Gene Ontology categories. The identified SSRs here may be a valuable addition for genetic mapping and future studies in phylogenetic analyses and genomic evolution.

## 1. Introduction

Cassava (*Manihot esculenta* Crantz) is one of the most important crops worldwide in terms of production and it is considered the most important food source for people living in tropical regions with arid soils [1]. World production of cassava was estimated to be 276 millions of tons in 2013 and therefore it is considered to be the eighth most important product [2]. Cassava is Euphorbiaceae and belongs to the genus* Manihot*, which contains 90 species approximately [3]. Cassava is a high-yielding crop and its roots constitute a major food source for over 800 million people [4–6] mainly from Africa, Asia, and South America [2]. Starch stored in cassava roots represents more than 80% of their dry weight [7]. This starch is transformed to be used in different industrial processes, in derivates such as alcohol and fructose-glucose syrups [8]. Cassava leaves provide proteins and vitamins A and B, mainly to African population [9]. This crop has high conversion rates of solar energy into carbohydrates and shows high tolerance to adverse abiotic stress [6]. Cassava plants can survive after long periods of drought in arid and low fertility soils [10].

Microsatellites (also known as* short tandem repeats* (STRs),* simple sequence repeats* (SSRs) [11, 12],* simple sequence length polymorphism* (SSLP), and* sequence tagged microsatellite site* (STMS)) [13] are sequence motifs of one to six bp, repeated in tandem. SSRs are widely spread in eukaryotic genomes [14] and are present even in organelle [15]. SSRs have been employed for studies of diversity [10, 16–18], phylogeny [19], and evolution [20], as molecular markers in marker-assisted selection [21] and have contributed significantly to the construction of genetic linkage maps [15, 22]. SSRs are considered allele specific [23], highly polymorphic, codominant [24], heterozygous, reproducible, economic [25], and multiallelic molecular markers [26]. The SSRs offer the opportunity to be employed in different studies given that they are under neutral selection when located in noncoding regions [27]. SSRs can be classified in two classes: class I is composed of those with ≥20 bp repeats and class II grouped SSRs from 12 to 20 bp. This classification is based on the observation that larger SSRs (class I) are demonstrated as more polymorphic than the shorter SSRs (class II) [28]. Changes in length are due to a replication phenomenon known as “slippage” [12], although the unequal crossover in recombination also has a significant influence [29]. SSRs have been considered as robust markers and have been transferred between different species [13]. These markers are relatively easy to automate [15] and are generally considered more informative than other markers such as the single nucleotide polymorphisms (SNPs) due to the number of alleles that can be detected [30]. SSRs have the advantage of being PCR-based markers because the flanking sequences are suitable for primer design [25].

SSRs could be functionally implicated in chromatin organization, gene expression, and recombination hotspots and could affect DNA replication [29]. SSRs are important for genome evolution as they constitute an important source of variation [31]. Furthermore, in some cases position and changes in SSRs are associated with phenotypic changes [20]. Despite their importance and wide usefulness, SSRs genomic distribution studies in plant species are relatively scarce [32].

With the advent and new advances in sequencing technologies, it is possible to analyze whole plant genomes for SSR discovery. Genomic studies of SSRs distribution have been conducted on* Arabidopsis* where it was first found that coding regions have a low frequency of SSRs and that these regions are highly rich in trinucleotides and hexanucleotides. These analyses also led to the conclusion that 5′ sequences had higher frequencies of SSRs than other genome sequences [20, 33] and that selective pressure acts differentially across genomic regions. An important feature is that in* Arabidopsis* there is high prevalence of A-rich repeats [33].

Molecular markers have been of paramount importance in cassava for genetic diversity [17, 34–39], evolution, and molecular systematic studies [10]. SSRs in cassava have been favored over DArTs (diversity arrays technology) due to their codominant and multiallelic nature [40]. Strategies for SSRs identification in cassava have included enriched DNA libraries [25, 34] and the pursuit in ESTs sequences [10, 23]. Given that multiple groups have identified SSRs markers independently, it is highly probable that the same markers have been found several times and named differently [30]. Genomic analyses of cassava SSRs would contribute to the understanding of cassava genome architecture and evolution and possibly correlate SSR's frequency, distribution, and sequence motifs, with genomic localization and function. We searched the cassava genome near-complete sequence (http://www.phytozome.com/) to gain an insight into genomic composition of cassava's SSRs. We carried out SSRs identification and characterization on the cassava's genome and their distribution in exons, introns, and UTR (untranslated regions). A Gene Ontology (GO) annotation was conducted for the SSRs present in the gene regions.

## 2. Materials and Methods

### 2.1. Sequences Gathering and SSRs Mining

Cassava whole genome sequence (version Cassava4) was obtained from the Phytozome database available at http://www.phytozome.net/cassava [41]. This 532.5 Mbp cassava genome sequence belongs to the genotype AM560-2, an inbred lined derived from the cultivar MCOL1505 [41]. SSRs identification was made with the Pearl script MISA (*MIcroSAtellite* identification tool, http://pgrc.ipk-gatersleben.de/misa). The parameters established for MISA were adjusted for the identification of class I SSRs (length ≥ 20 bp) of di-, tri-, tetra-, penta-, and hexanucleotides. Class I SSRs were chosen because they have proven to be more polymorphic than SSRs of 12 to 20 bp [28]. Mononucleotides were not considered because of the possibility of sequencing or assembly errors [42]. For compound SSRs (distinct and adjacent SSRs), the maximum difference between two SSRs was set as 100 bp or less. For comparative purposes, a genomic identification of SSRs in other species was also done using the same parameters described above. The genomes of the related species selected were:* Populus trichocarpa* (poplar) and* Linum usitatissimum* (flax) which belongs to the order Malpighiales;* Ricinus communis* (*Ricinus*) [43] and* Jatropha curcas* (*Jatropha*) [44] that belong to the Euphorbiaceae family.

Coding, 3′ UTR, and 5′ UTR cassava sequences were extracted using the Biomart tool [45] and introns were extracted using a Pearl script. Altogether we obtained ~40 Mbp of coding sequences, ~50 Mbp of intron sequences, and ~2 Mbp and ~4 Mbp of 5′ and 3′ UTR sequences, respectively. SSRs density, SSR types, and motif distribution in cassava were assessed, analyzed, and compared through information stored in Excel files.

### 2.2. Codon Usage Analysis and Functional Categories of Genes

With the aim of obtaining the codon usage in cassava coding sequences we used the CUSP program of EMBOSS [46] (The European Molecular Biology Open Software Suite, Cambridge, UK; http://emboss.sourceforge.net/).

For the purpose of assigning functional categories to the sequences from the different gene regions containing SSRs, we searched for the classes to which each sequence belonged and were grouped according to GO categories. The functional classes for each gene were obtained using the Biomart data mining tool hosted in Phytozome. The CateGOrizer tool (http://www.animalgenome.org/bioinfo/tools/countgo/) was used to count GO classes and group them into functional categories. The GO_ROOT classification method and single counting method were set as parameters for the GO terms counting, to obtain a classification based on the three main categories: molecular function, biological process, and cellular component. Plant_GOslim classification method and single counting method were used as parameters to group the sequences in the different subcategories [47].

## 3. Results

### 3.1. Whole Genome SSRs Density Comparison

**Figure 1:**
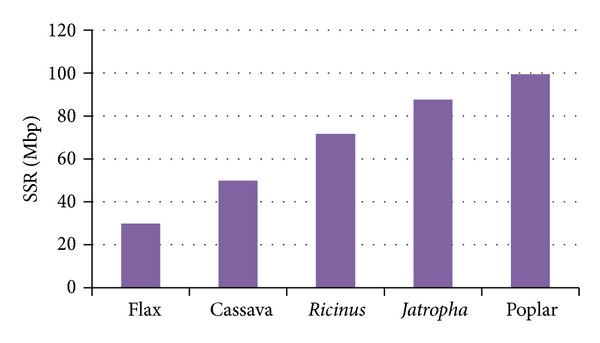
SSR density comparison between genomes from several plant species.

In order to conduct an exploratory analysis of the SSRs present in the complete cassava genome and to make comparisons with genomes of related species, we detect 26.579 class I SSRs in the cassava genome, using the MISA tool. Considering the whole genome sequence length (536 Mbp), the density of SSRs present in cassava was estimated to be ~50 SSRs per Mbp (Figure 1). In* Ricinus* a density of 71,7 SSRs/Mbp was identified, while in poplar we found 99 SSRs/Mbp. The SSRs density was 30 SSRs/Mbp for flax and 87,7 SSRs/Mbp in* Jatropha*. In general, the SSRs density in cassava was less than the average found in the assessed species (67,7 SSRs/Mbp).

**Figure 2:**
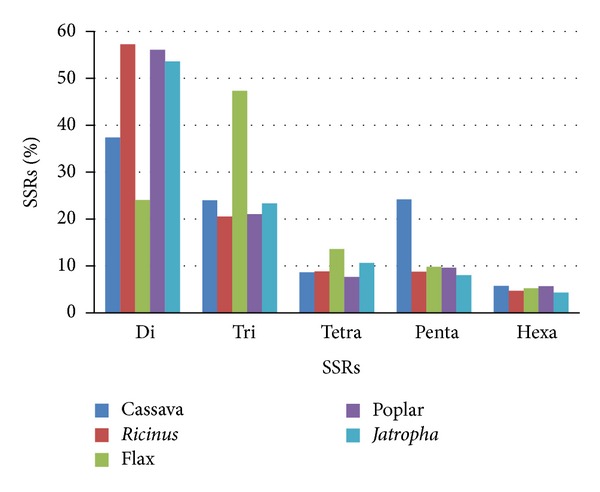
Frequency (%) of the number of each motif of identified SSRs in the assessed species.

Based on the type of repetition we found that 37,4% of all SSRs found in cassava correspond to dinucleotides, 24% are trinucleotides, 8,6% are tetranucleotides, 24,2% pentanucleotides, and 5,8 correspond to hexanucleotides (Figure 2). Most SSRs in cassava genome are dinucleotides as have been observed in most species [15]. Indeed in most of the evaluated species we observed that the most common SSR type is dinucleotide with the exception of flax, which has a higher number of trinucleotides. According to this, in flax dinucleotides just accounted for 24%, while trinucleotide accounted for 47,3% of all SSRs identified. A high proportion of pentanucleotides was found in cassava (24,2%) in contrast to the other species, which have an average of 9% of this type of SSR.

### 3.2. Distribution of SSRs in Different Genomic Regions

**Figure 3:**
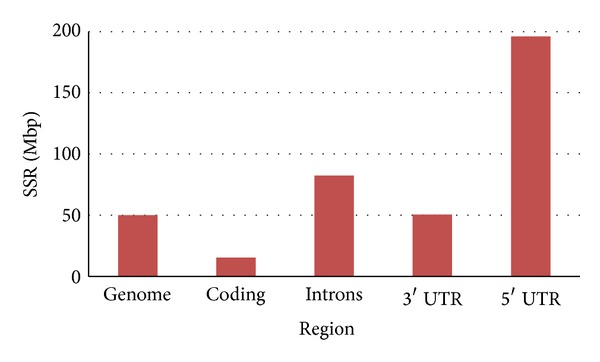
SSR density in different cassava genome regions.

To determine the distribution of SSRs in the cassava genome, we carried out an SSR search in coding, UTRs, and intron sequences. For this purpose we extracted sequences corresponding to each of these regions. We obtained coding sequences corresponding to 34.151 annotated genes, 3′ UTR sequences from 15.420 genes and 5′ UTR sequences from 14.111 genes. The low number of genes having UTRs is due to deficient gene annotation. A Pearl script allowed the extraction of 122.806 intron sequences corresponding to 24.309 genes. Following the SSRs search on each of these regions, as we expected, coding regions were found to have the lowest density of SSRs (Figure 3). We found that the average density of SSRs in the whole genome is higher than in coding regions. For example we identified 49,9 SSRs/Mbp SSRs in the whole genome while only 15,5 SSRs/Mbp were found in coding sequences. Of 34.151 coding sequences analyzed, we found that 587 contained at least one SSR and 32 had more than one SSR. The density of SSRs in the whole genome was lower than in noncoding regions (introns and UTRs) (Figure 3). According to a previous report, which have indicated that UTR regions are SSR rich [48], we observed that in cassava 5′ UTRs contain the greatest amount of SSRs; we identified 434 (196,1 SSRs/Mbp) SSRs in 5′ UTRs and 202 (50,5 SSRs/Mbp) SSRs in 3′ UTRs. We expected to find more SSRs in the 3′ UTR than in the 5′ UTRs as it has been reported previously [48]. However, we observed that 5′ UTR sequences have between 2,4- and 12,6-fold higher SSR density than other regions and almost fourfold higher density than in the whole genome. Higher SSRs densities in 5′ UTRs were also observed in* Arabidopsis* and a similar proportion to that we found was identified in rice [20]. Finally we identified 82,3 SSRs/Mbp in introns sequences. The existence of more than one SSR in a single sequence was found to be scarce. Only 0,09% of coding sequences, 0,39% of introns, 0,16% of 5′ UTR, and 0,02% of 3′ UTR have more than one SSR in a single sequence.

### 3.3. SSRs Motifs in Different Regions of the Genome

**Figure 4:**
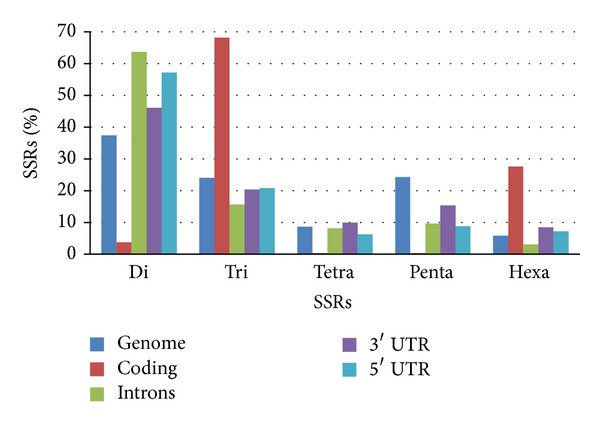
Frequency (%) of the number of each SSR motif identified on different cassava genome regions.

A comparison of the motifs in different genomic regions was done because the motifs proportion changes across the genome in a similar manner to the SSRs number. As a result of selection pressure it has been noted that most of the SSRs found in coding regions are tri- or hexanucleotides avoiding frame shifts in this way [20, 49, 50]. This situation was also found in cassava coding sequences where tri- and hexanucleotides account for 95,6% of the SSRs and almost no tetra- and pentanucleotides were identified on these regions. The results also suggest that noncoding sequences, as observed in the whole genome, have a high proportion of pentanucleotides (Figure 4).

**Figure 5:**
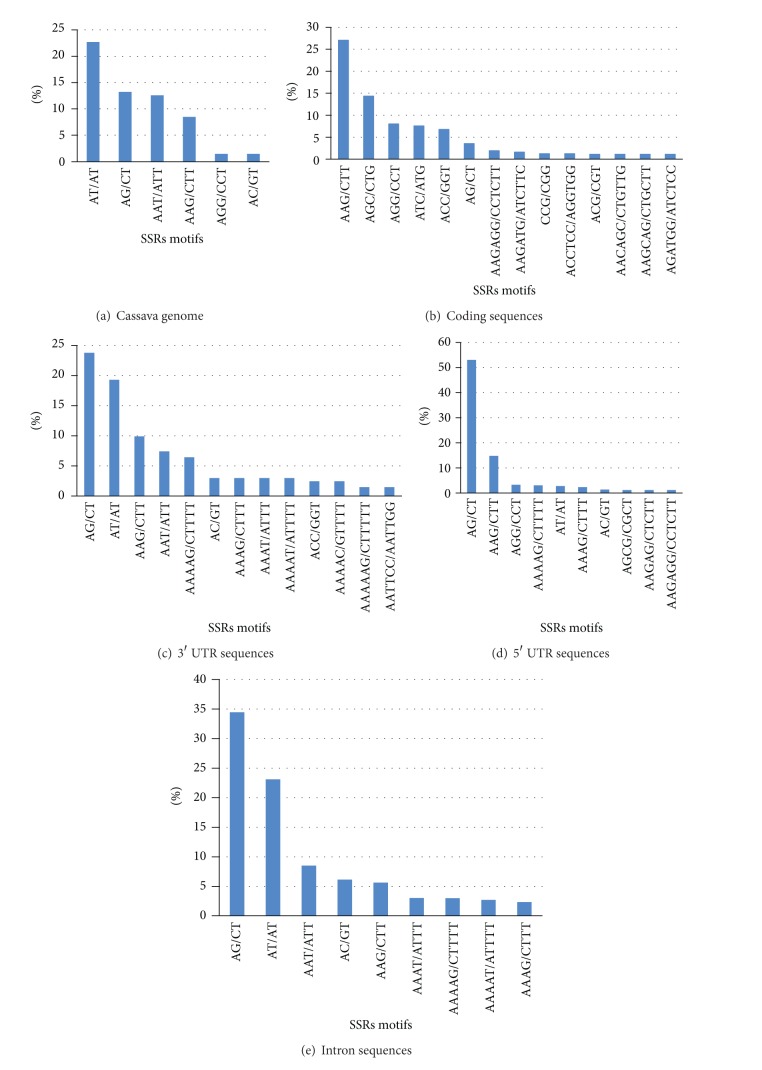
Frequency in percentage of the number of each SSR motif in (a) cassava genome, (b) coding sequences, (c) 3′ UTR sequences, (d) 5′ UTR sequences, and (e) introns.

The type of motif present in each region of the genome was analyzed. Only motifs present with a frequency of 1% or more were considered. In general there was a high prevalence of A-rich repeats. This type of SSR may have evolved from polyA stretches and could generate important secondary structures [33]. In the entire cassava genome there is prevalence of the AT/AT motif represented by approximately 22% of the SSRs identified (Figure 5(a)). This is the most abundant motif found in several plant genomes [12, 15, 26, 48, 51]. In cassava coding sequences we found a prevalence of AAG/CTT and AGC/CTG (Figure 5(b)). SSRs in coding regions could give an indication of codon usage preference [52]. To determine if the SSRs identified in coding regions correspond to the most used triplets in cassava, we carried out a codon usage analysis. The motif AAG which is the most commonly found in coding sequences is in fact the third most used codon in cassava with a percentage of 3,2% among all the nucleotide combination triplets (Table 1). The SSR motifs AGC/CTG, AGG/CCT, ATC/ATG, and ACC/GGT that were also found in high frequencies are used in percentages between 0,9 and 1,4% indicating that these codons are not used frequently.

In noncoding sequences the most common SSR motif is AG/CT (Figures 5(c), 5(d), and 5(e)) similar to previous reports for several plant species [27, 48, 53]. In 5′ UTR sequences, most of the SSRs were of the AG/CT and AAG/CTT type. Similar observations were reported in other dicotyledonous species like* Arabidopsis* and soybean, but not in monocot plants such as rice or maize [33]. These differences in motif distribution in upstream gene sequences often lead to differences in genomic structure and gene regulation on both groups of plants [20, 49, 54]. The GC type was not identified in any of the cassava sequences groups.

### 3.4. Gene Description Analysis according to Gene Ontology Categories

**Figure 6:**
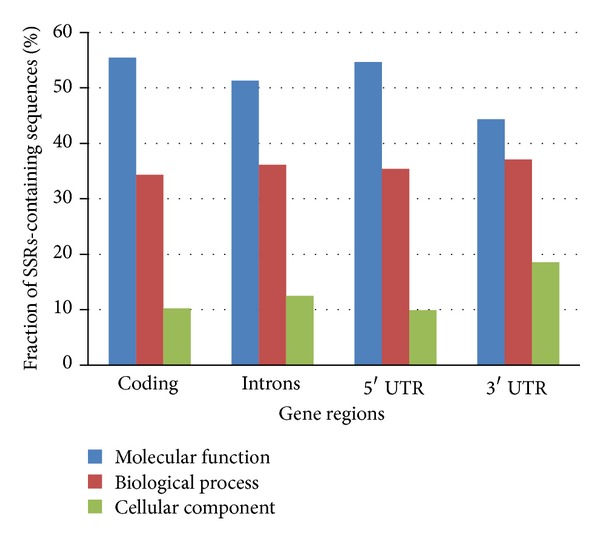
Classification of the sequences of the different cassava gene regions that contain SSRs, according to the three main categories from GO.

In order to gain some insight into the putative function of the genes containing SSRs, we classified those genes according to GO categories (Figure 6). Putative molecular function was attributed to 55,4% of gene coding sequences that contain SSRs, 51,3% of intronic sequences, and 54,7% and 44,3% of 3′ and 5′ UTR sequences, respectively. About 35,7% of the sequences containing SSRs belonged to genes classed in biological processes, while 10,2%, 12,5%, 9,9%, and 18,7% of the coding, intronic, and 3′ and 5′ UTR sequences, respectively, corresponded to genes grouped in the cellular component category.

A detailed categorization for each different GO category was made (Supplementary Figures  1, 2, 3, and 4 in Supplementary Material available online at http://dx.doi.org/10.1155/2014/471461). When the subcategories comprised in “biological process” were compared, we observed that although there were some evident differences, the majority of genes containing SSRs belonged to the cellular process subcategory. The metabolic, biosynthetic, and protein metabolic processes were the categories with more SSR-containing genes. In terms of the cellular component, a common feature was that sequences containing SSRs belonged to genes that encoded proteins located frequently inside cell, cell membrane, and nucleus. Regarding the molecular function, the SSRs-containing genes were mostly related to catalytic, binding, hidrolase, transferase, and transporter activity.

Interestingly we found that cassava genes coding for proteins located in thylakoid, vacuole, and Golgi apparatus contain SSRs exclusively on intron sequences. A similar situation was observed for genes related to carbohydrate binding, pollen-pistil interaction, pollination, regulation of gene expression, epigenetic process, and reproduction. We also observed that there is just one gene with SSR associated with embryonic development and its SSR is located on the 5′ UTR of the gene. In addition some genes contain SSRs in their coding and intron sequences but not in the UTR regions (related to carbohydrate metabolic processes, lipid binding, motor activity, and genes that encode proteins located on the cell wall, external encapsulating structures, and peroxisomes). In a similar manner, genes related to response to biotic stimulus have SSRs exclusively on the coding and intron sequences. On the other hand genes related to response to abiotic stimulus had SSRs exclusively on their UTR sequences. For genes associated with response to endogenous stimuli and stress response, SSRs were detected in all their regions (intron, coding, and UTRs sequences).

## 4. Discussion

In this work we identified and analyzed the SSRs present in the cassava genome. We found that, as expected, cassava has high frequencies of dinucleotides and that a unique feature of this plant was its unusual high frequency of pentanucleotides. The predominance of a specific SSR class has been observed in rice [55]. In terms of gene analysis, coding sequences are the regions with the lowest density of SSRs while the 5′ UTRs are the counterpart with the highest content. In general we identified 621 SSRs (15.5 SSRs/Mbp) in coding sequences, 4.120 SSRs (82,3 SSRs/Mbp) in introns, and 434 (196,1 SSRs/Mbp) and 202 (50,5 SSRs/Mbp) SSRs in 5′ and 3′ UTR sequences, respectively.

It is estimated that in cassava there are approximately 1.000 SSRs previously identified. After following the methodology proposed here we identified 26.579 SSRs in the cassava genome. Previous studies on SSR detection in cassava genomic libraries reported the identification of 12 [3], 32 [34], 545 [25], or 1.576 SSRs [22]. Previous efforts in searching cassava SSRs in sequences yielded the identification of 531 [56], 49 [57], 836 [23], 1.889 [10], 431 [4], 7.270 [58], or 163 SSRs in cassava ESTs. The low number of SSRs previously found could be explained for the low number of genome sequences reported at that moment. With the recent cassava genome release a global and genomic analysis of SSRs is possible. This is the first report of SSR analysis in the whole genome of cassava. Additionally, no study had focused earlier on the distribution of SSRs along different gene regions in cassava. The identification of SSRs in several gene sequences is not only informative but also useful to develop makers to map the genes in which they reside. Here we report 4.747 SSRs within genes. However it is important to note that some of these could correspond to anonymous, with an unknown function, type of markers and although they have been useful for developing genetic maps and for diversity studies most of them have no specific known function.

Through SSRs data comparison we determined that cassava has only 49,9 SSRs/Mbp being one of the species with lower SSRs densities in its genome compared with phylogenetically closer species. Based on cassava nuclear DNA quantity it has been estimated that the cassava genome is 772 Mbp [59] and nonetheless the sequenced genome is 533 Mbp. The lacking sequences of about ~240 Mb could consist of repetitive DNA which has not been assembled [41]. Although it has been observed that SSRs are preferentially found in nonrepetitive DNA [48], the low quantity of SSRs in cassava could be associated with the possibility that the nonassembled sequences in the genome would have a considerable amount of SSRs.

It is important to note that SSRs identified in gene sequences are potential powerful molecular markers for use in breeding programs. Due to their location inside genes, these markers save effort and resources in the early stages of searching for markers closely linked to particular genes. In addition they can be employed in association mapping studies. The SSR markers identified in this work would be an important resource for genetic mapping analysis of the genes in which they are located. In addition, they could help to make phylogenetic analysis to understand the diversity of those genes. Owing to their mutation properties, these SSRs would give hints about evolutionary changes on the cassava genome.

The prevalence of dinucleotide AG/CT in cassava has been observed in previous studies where the search was made through the development of SSRs libraries [25, 34] or by* in silico* SSR search in ESTs [10, 56]. Here we observed that this is the second most observed motif in the genome and it is the most frequent in noncoding sequences. The latest result is consistent with the statement that the AG/CT is in fact the most common dinucleotide in vascular plant ESTs [52, 53] and in coding regions according to Morgante et al. [48]. We observed that in cassava the dinucleotide AC/GT or GA/CT accounted only for 1,5% of the SSRs in all the evaluated sequences, while the other dinucleotides added up to 38%. Compared to the genomes of animals, it is considered that the repeats AC/GT are scarce in plants [48]. Additionally, no GC/CG dinucleotides were found. Previous studies highlighted the absence of this motif in sequences of several plant species [51, 53] and are indeed the least frequent SSR in almost every assessed organism with the exception of* Escherichia coli* [60].

The availability of cassava genome sequences enabled a more effective assessment of SSR marker distribution in this study. This is important because correlation between cassava physical and genetic map can now be made. This will be quite useful in cassava since the genome sequence is highly fragmented. Finally the GO categories assignment of the genes where the SSRs were identified can be useful in studies where the objective is to map a specific group of genes corresponding to a functional category such as abiotic or biotic stress.

## Supplementary Material

The supplementary material includes figures that show the number of sequences, for each gene region (coding, intrón and UTR), that are associated to GO detailed categories. These GO categories belong to one of the three GO subcategories; biological process, cellular component and molecular function.

## Figures and Tables

**Table 1:** Codon usage in cassava.

Codon	Amino acid	Percentage	Number of codons	Codon	Amino acid	Percentage	Number of codons
GAT	D	3,8	507.698	GAC	D	1,4	190.687
GAA	E	3,5	470.113	AGG	R	1,4	182.442
AAG	K	3,2	425.450	TTA	L	1,3	177.948
AAT	N	3,0	396.685	ATC	I	1,3	176.053
AAA	K	3,0	393.592	CTG	L	1,3	171.523
GAG	E	2,9	391.255	TGG	W	1,3	169.733
GCT	A	2,8	379.781	GGG	G	1,2	164.900
ATT	I	2,7	357.473	CTC	L	1,2	159.942
GTT	V	2,6	349.383	AGC	S	1,2	156.337
TTT	F	2,5	338.924	TCC	S	1,2	155.279
TCT	S	2,5	335.970	GCC	A	1,1	147.095
CTT	L	2,5	330.282	GGC	G	1,1	146.652
TTG	L	2,4	324.968	GTA	V	1,1	143.123
ATG	M	2,4	320.621	TAC	Y	1,1	140.126
GCA	A	2,4	316.233	TGT	C	1,0	135.360
GGA	G	2,2	292.859	GTC	V	1,0	129.696
TCA	S	2,1	285.008	CTA	L	1,0	128.518
CAA	Q	2,0	271.535	AAG	T	0,9	123.476
CCT	P	2,0	262.772	TGC	C	0,8	111.835
GGT	G	2,0	260.909	CAC	H	0,8	104.586
CCA	P	1,9	249.958	CCC	P	0,7	89.657
ACT	T	1,8	244.189	CGT	R	0,6	84.020
AGA	R	1,8	237.036	CGA	R	0,6	77.708
TAT	Y	1,8	236.102	TCG	S	0,5	66.696
GTG	V	1,7	229.620	GCG	A	0,4	57.365
TTC	F	1,7	228.875	CGG	R	0,4	55.618
CAG	Q	1,7	227.650	CGC	R	0,4	55.069
ACA	T	1,6	217.490	CCG	P	0,4	52.497
CAT	H	1,6	212.034	ACG	T	0,4	51.232
AAC	N	1,6	207.756	TGA	∗	0,1	13.438
AGT	S	1,5	203.223	TAA	∗	0,1	10.443
ATA	I	1,5	193.364	TAG	∗	0,1	7.536
