# Supplementary material for: In Silico Genome Comparison and Distribution Analysis of Simple Sequences Repeats in Cassava
Source: Int J Genomics. 2014 Oct 13;2014:471461. doi: 10.1155/2014/471461 (PMC4211302; doi:10.1155/2014/471461)
Supplement: Supplementary file 1 — The supplementary material includes figures that show the number of sequences, for each gene region (coding, intrón and UTR), that are associated to GO detailed categories. These GO categories belong to one of the three GO subcategories; biological process, cellular component and molecular function. [file 471461.f1.pdf]

Supplementary figure 1: Number of coding sequences in each GO subcategory for a) biological process, b) cellular component category and c) molecular function.

a)

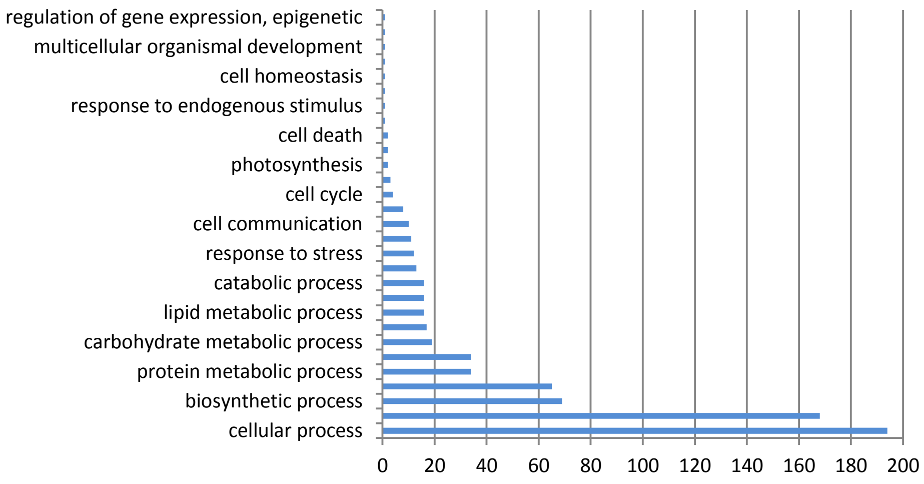

b)

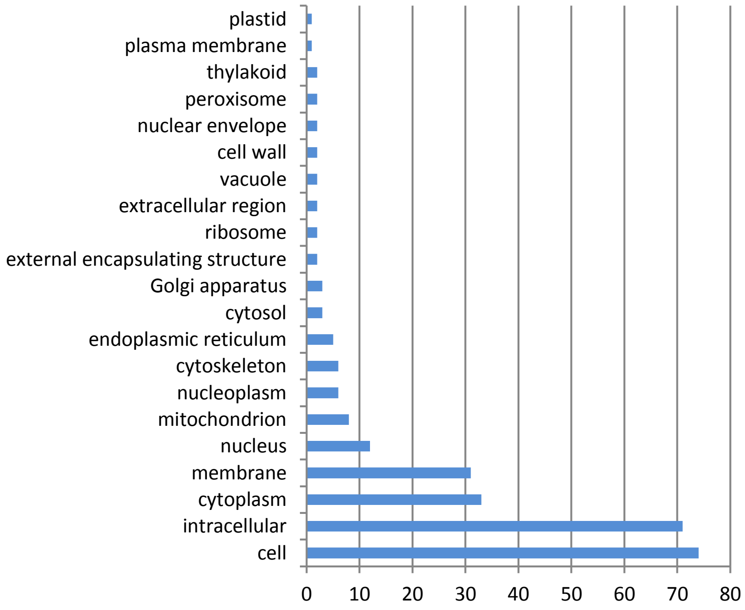

c)

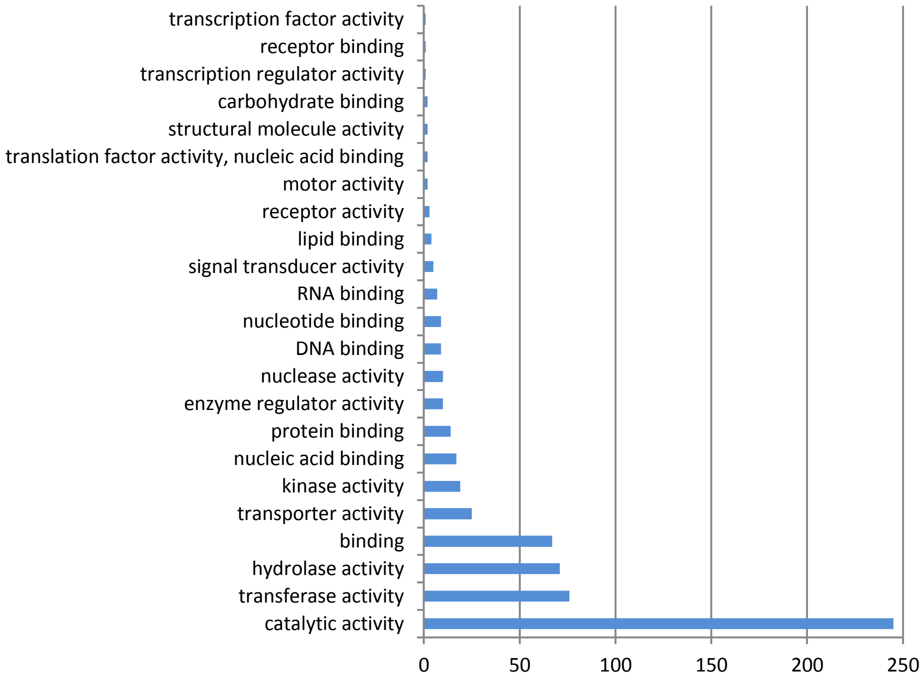
transcription factor activity
receptor binding
transcription regulator activity
carbohydrate binding
structural molecule activity
translation factor activity, nucleic acid binding
motor activity
receptor activity
lipid binding
signal transducer activity
RNA binding
nucleotide binding
DNA binding
nuclease activity
enzyme regulator activity
protein binding
nucleic acid binding
kinase activity
transporter activity
binding
hydrolase activity
transferase activity
catalytic activity
0
50
100
150
200
250

Supplementary figure 2: Number of intron sequences in each GO subcategory for a) biological process, b) cellular component category and c) molecular function.

a)

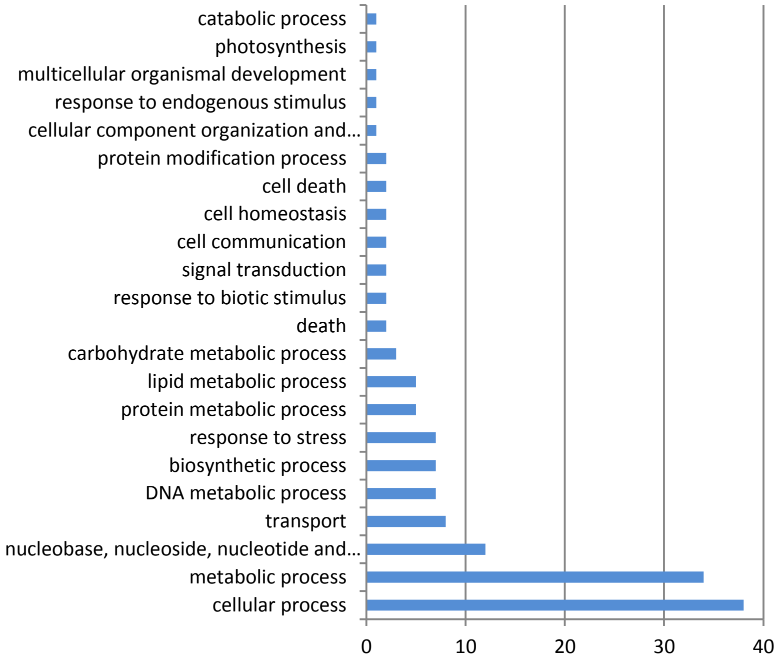

b)

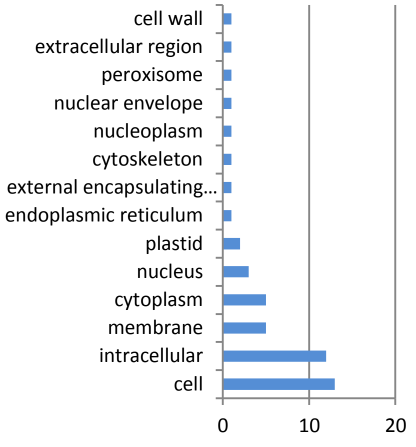

c)

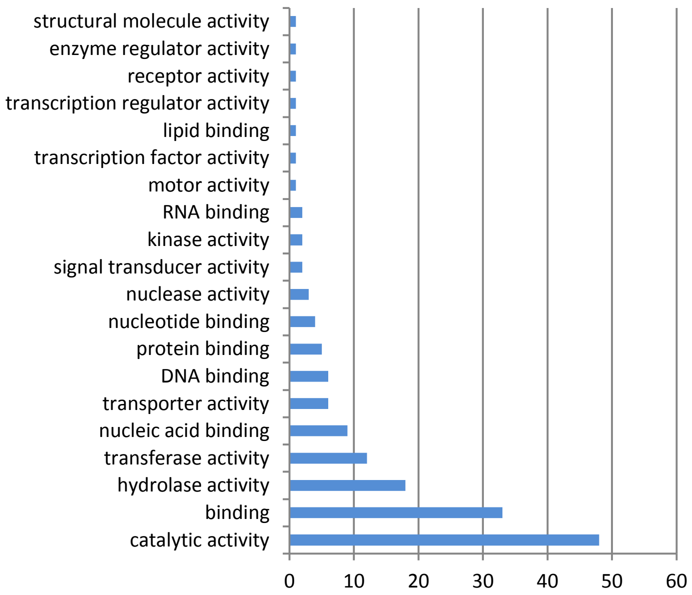
structural molecule activity
enzyme regulator activity
receptor activity
transcription regulator activity
lipid binding
transcription factor activity
motor activity
RNA binding
kinase activity
signal transducer activity
nuclease activity
nucleotide binding
protein binding
DNA binding
transporter activity
nucleic acid binding
transferase activity
hydrolase activity
binding
catalytic activity
0
10
20
30
40
50
60

Supplementary figure 3: Number of the 5'UTRs sequences in each GO subcategory a) biological process, b) cellular component category and c) molecular function.

a)

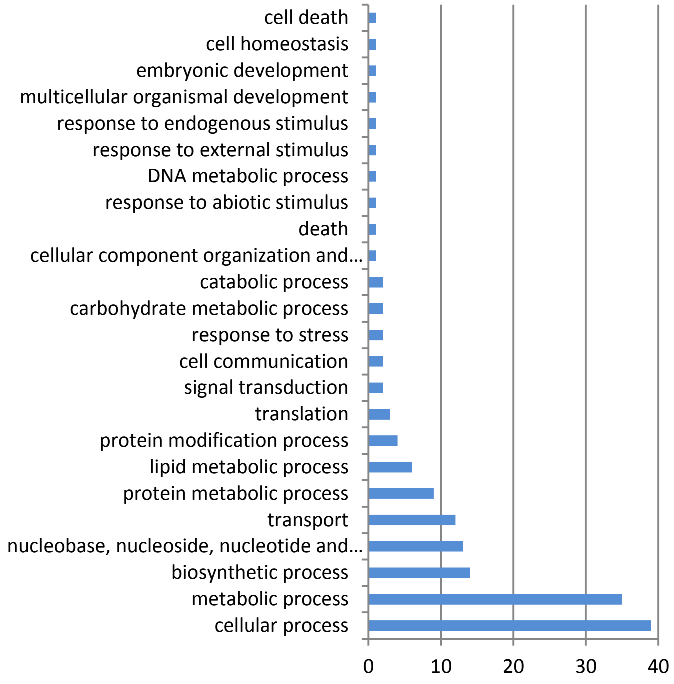

b)

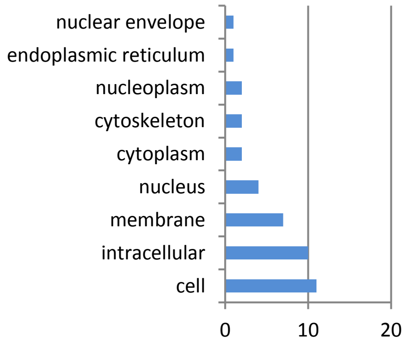

c)

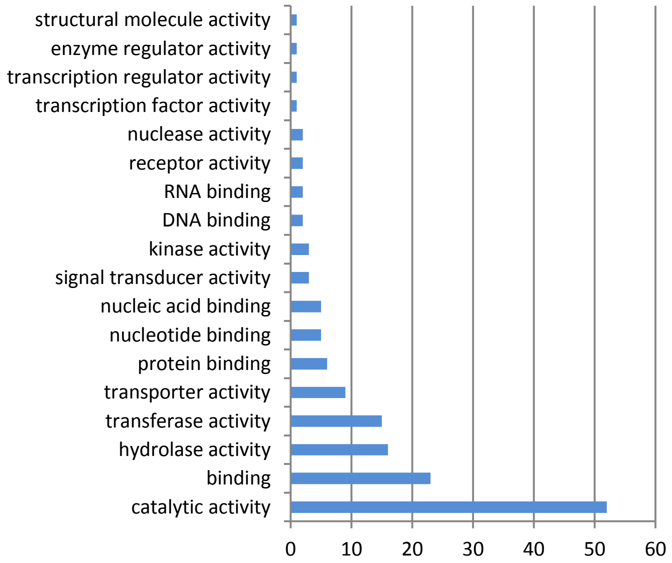

structural molecule activity
enzyme regulator activity
transcription regulator activity
transcription factor activity
nuclease activity
receptor activity
RNA binding
DNA binding
kinase activity
signal transducer activity
nucleic acid binding
nucleotide binding
protein binding
transporter activity
transferase activity
hydrolase activity
binding
catalytic activity
0
10
20
30
40
50
60

Supplementary figure 4: Number of 3’ UTRs sequences in each GO subcategory for a) biological process, b) cellular component category and c) molecular function.

a)

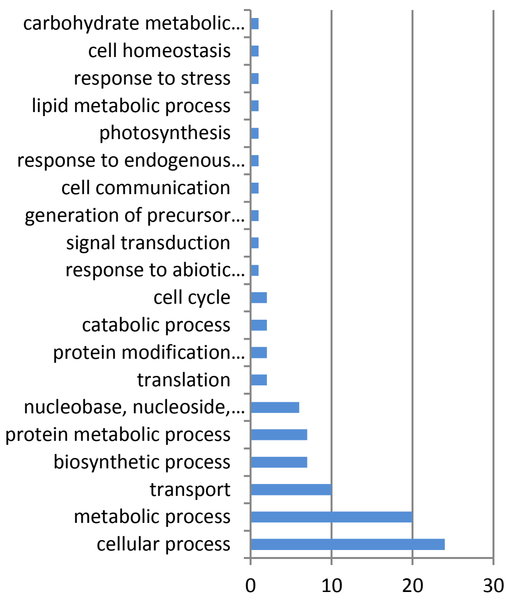

b)

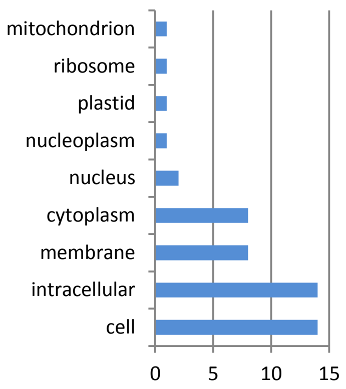

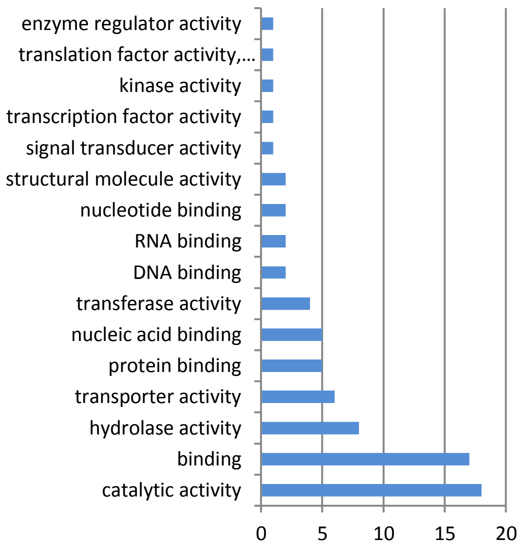

c)
